# The RoxyScan is a novel measurement of red blood cell deformability under oxidative and shear stress

**DOI:** 10.1038/s41598-024-56814-8

**Published:** 2024-03-15

**Authors:** Sandra K. Larkin, Carolina Hernández, Eduard J. van Beers, Richard van Wijk, Frans A. Kuypers

**Affiliations:** 1 Department of Pediatrics, University of California San Francisco, UCSF RBClab, 5700 MLK Jr. Way, Oakland, CA 94609 USA; 2 Department Central Diagnostic Laboratory - Research, University Medical Center Utrecht, Utrecht University, 3584 CX Utrecht, The Netherlands; 3 Center for Benign Hematology, Thrombosis and Hemostasis - Van Creveldkliniek, University Medical Center Utrecht, Utrecht University, 3584 CX Utrecht, The Netherlands

**Keywords:** Biological techniques, Medical research

## Abstract

Exposure to both oxidative and shear stress, a condition that the red blood cell (RBC) continuously experiences in the circulation in vivo can be mimicked in a Couette type viscometer and monitored by ektacytometry. RBCs maintain their deformation and orientation under shear stress and oxidative stress until a threshold is reached at which these conditions appear to overwhelm the elaborate and complex pathways that maintain a proper redox environment in the cell. Oxidative stress under shear alters the ability of the cell to deform, changes cell morphology, its orientation in the shear stress field, and appears to alter intracellular and membrane characteristics. The application of the RoxyScan technology allows the comparison of oxidant effects and the role of antioxidant systems. This provides the opportunity to study the ability of RBC to deal with oxidative stress in various conditions, including RBC disorders such as sickle cell disease (SCD).

## Introduction

Red blood cells (RBC) have a limited lifespan as they transport oxygen from the lungs to the tissues. Reactive oxygen species (ROS) challenge the viability of RBC by disrupting membrane properties and cellular deformability^1^. A complex arsenal protects RBC and renders them a sink for oxidative stress^2,3^. Glycolysis provides the energy for the intricate system that preserves the redox state of RBC, maintains membrane integrity, and keeps hemoglobin in its ferrous state. The systems that repair and maintain membrane viability are also targets for oxidant damage, and since RBC are not capable of de-novo synthesis of proteins and lipids, damaged RBC are removed from the circulation to maintain the proper physiological function of blood. RBC replacement is significantly higher for RBC less able to maintain a proper redox status such as observed in patients with sickle cell disease (SCD) and thalassemia. We argue that assessment of the deformability under shear and oxidative stress, would provide insight into the ability of RBC to maintain redox status and functionality in the circulation. RoxyScan ektacytometry using the LoRRca MaxSis^4^ exposes RBC to oxidant and shear stress. We demonstrate that the elongation index (EI) measured using the ektacytometric laser diffraction pattern decreases as the result of oxidative stress, relates to the changes in morphology and orientation of RBC in the field of shear stress, and reports on the ability of RBC to maintain membrane viability under oxidative stress. The oxidants used here include tert-butyl hydroperoxide (tBOOH) as an example of a more water-soluble oxidant and cumene hydroperoxide (CuOOH) as an oxidant that targets a more hydrophobic environment (membrane). Neither of these chemical oxidants will occur in vivo but represent free radical species that target either the RBC cytosol or membrane. We also tested hydrogen peroxide (H_2_O_2_) as a naturally occurring oxidant in the RBC.

## Results

**Figure 1:**
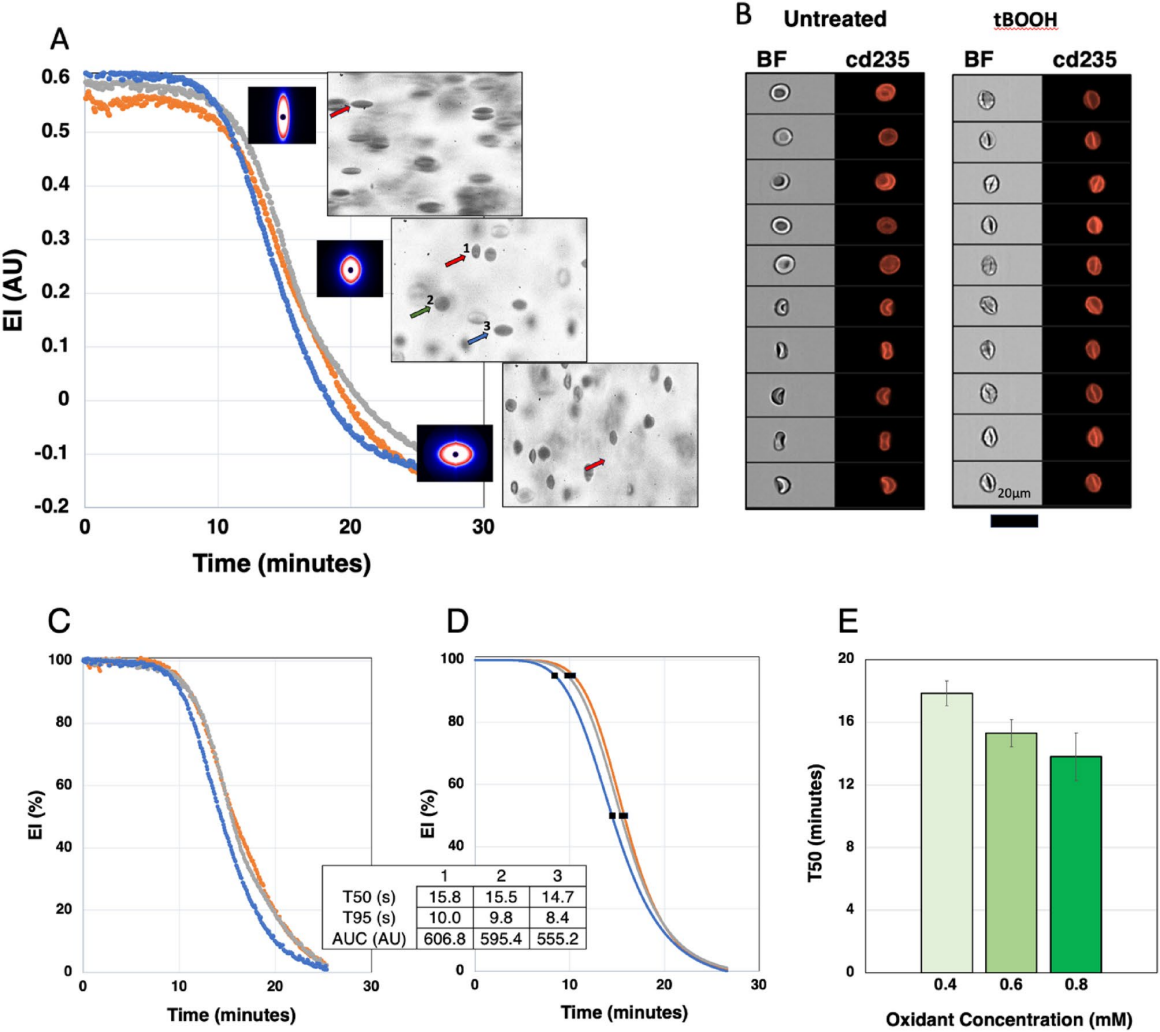
RoxyScan results with diffraction patterns, video microscope images, and ImageStream images. (**A**) Example curves of normal RBC at 50 × 10^6^/ml in the viscometer, from 3 donors incubated with 0.6 mM tBOOH under 30 Pa shear stress. The inserted pictures with the black background show the diffraction pattern at different times. Microscopic video sections at these timepoints show the shape of the cells in the viscometer. The arrows indicate different shapes further described in the text. (**B**) Examples of the morphology of the RBC before the RoxyScan assay (untreated) and after the assay with 0.6 mM tBOOH showing the brightfield image in the first column (BF) and anti-cd235a antibody labelling of the membrane in the second column. (**C**) Normalized data of EI in time as percentage of the starting (EI start) value of EI. (**D**) Curves based on a sigmoid curve fit of the data in Fig. 1C and the derived times when EI dropped to respectively 95% (T95) or 50% (T50) of the starting value as well as the area under the curve (AUC) for the three samples. (**E**) T50 of normal control samples (n = 3) tested at three concentrations: 0.4, 0.6, and 0.8 mM tBOOH.

The laser light passing through a population of millions of RBC on the viscometer results in a diffraction image that relates to the shape of the cells^5–7^. The LoRRca software fits the image of the diffraction pattern to an ellipsoid shape. The elongation index, or EI, is defined as (A − B)/(A + B) where A is the length of the vertical axis of the ellipsoid and B is the length of the horizontal axis. In the absence of shear, the diffraction pattern is round, A = B, and EI = 0. Increasing the shear will elongate the RBC and they orient horizontally in the field of shear stress and the diffraction pattern is vertical with A > B. At a shear stress of around 30 Pa the maximal deformation is reached. The pictures of the laser diffraction pattern in Fig. 1A show this elongated ellipsoid shape under the shear stress in the viscometer at the start of the incubation. Normal human RBC exposed to a shear stress of 30 Pa exhibit an EI of around 0.6 AU (supplementary Fig. 4). In the absence of oxidant, while keeping the shear stress at 30 Pa, the EI does not change during the 25–30 min in the viscometer (supplementary Fig. 1). In contrast, in the presence of 0.6 mM tBOOH, the diffraction pattern changes to a rounder shape after a few minutes and the EI starts to decrease. The EI decreases further and ultimately the diffraction pattern of cells shows a slight horizontal ellipsoid pattern with a negative EI as the vertical axis (A) becomes smaller than the horizontal axis (B) and (A − B)/(A + B) renders a negative value. This suggests that cells oriented differently in the path of the laser.

The microscopic video assessment, shown in Fig. 1A, shows cells in the viscometer that are ellipsoid shaped and vertically oriented (red arrow #1), round (green arrow #2), as well as less elongated (blue arrow #3). These data show that the changes in diffraction pattern and the value of EI reflects the diffraction pattern generated by a heterogeneous population of different deformed cells with various orientations in the field of shear. Whereas the EI is a good measure of the average assessment of millions of cells in the path of the laser, the microscopic video assessment is merely qualitative, given the low number of cells, but suggests an increasing number of vertically oriented ellipsoid shaped RBC that pivot around a vertical axis.

We assessed cell morphology before and after exposure to shear stress with tBOOH (Fig. 1B). Typical examples of bright field images of cells and anti-cd235a-PE fluorescence show the normal morphology of untreated cells compared to the images obtained after exposure to tBOOH showing most cells with a slightly elongated and folded morphology, resembling cells as observed with video microscopy that orient vertically in the viscometer. Cells removed from the viscometer after the final EI is reached followed by reintroduction in fresh PVP did not recover to normal morphology or the starting value of EI (data not shown), indicating that the damage to the RBC was irreversible. The ratio of the amount of oxidant to the number of RBC in the viscometer affects the decline in EI. As shown in supplementary Fig. 2, four different cell concentrations of the same blood sample were incubated under the same oxidant and shear conditions. The number of RBC was determined using the Advia 2120 and different blood volumes were added to result in 25, 50, 75 or 100 million RBC/ml of PVP exposed to a final concentration of 0.6 mM tBOOH under the same shear. At the same oxidant concentration, an increase of RBC resulted in an increase of T50 while a decrease in RBC numbers decreased the T50. To standardize the experimental setup, all subsequent experiments used 50 million RBC/ml of PVP in the viscometer where they are exposed to oxidant at the same shear stress of 30 Pa.

To describe the change of EI in time, we used a computer-generated sigmoid curve fit of the data. The mathematical fit is based on the start EI, final EI, the steepness of the curve, and the halfway point of the EI between start and final value (T50). This approach rendered an excellent fit of the raw data, as shown in supplementary Fig. 1 for three different oxidants. This mathematical approach also provides the option to describe the change of EI over time in a normalized fashion with a start at 100% and a final at 0%. Figure 1C shows the same data as shown in Fig. 1A calculated as percentage in time with the corresponding sigmoid curve fit shown in Fig. 1D. As expected, either the time to drop 5% below the starting EI (T95), the T50, or AUC of the sigmoid fit can be used to compare different results. The T50 and the steepness of the curve do not change between raw and normalized data. The starting value of EI varied for each sample tested, and the final value of EI varied with the oxidant used. Of the three oxidants tested, tBOOH leads to a final negative EI value of around − 0.18 AU, CuOOH to an EI of − 0.1 AU and H_2_O_2_ to an EI just under 0 AU. At lower levels of oxidant stress the final level of EI may not be reached within a practical timeframe, despite an excellent curve fit based on the final EI for each oxidant. We decided to use 25–30 min as a practical time limit for each assay. The concentration dependence of the assay is shown in Fig. 1E. Different blood samples from the same normal control donor were processed on three different days at three concentrations 0.4, 0.6, and 0.8 mM tBOOH.

**Figure 2:**
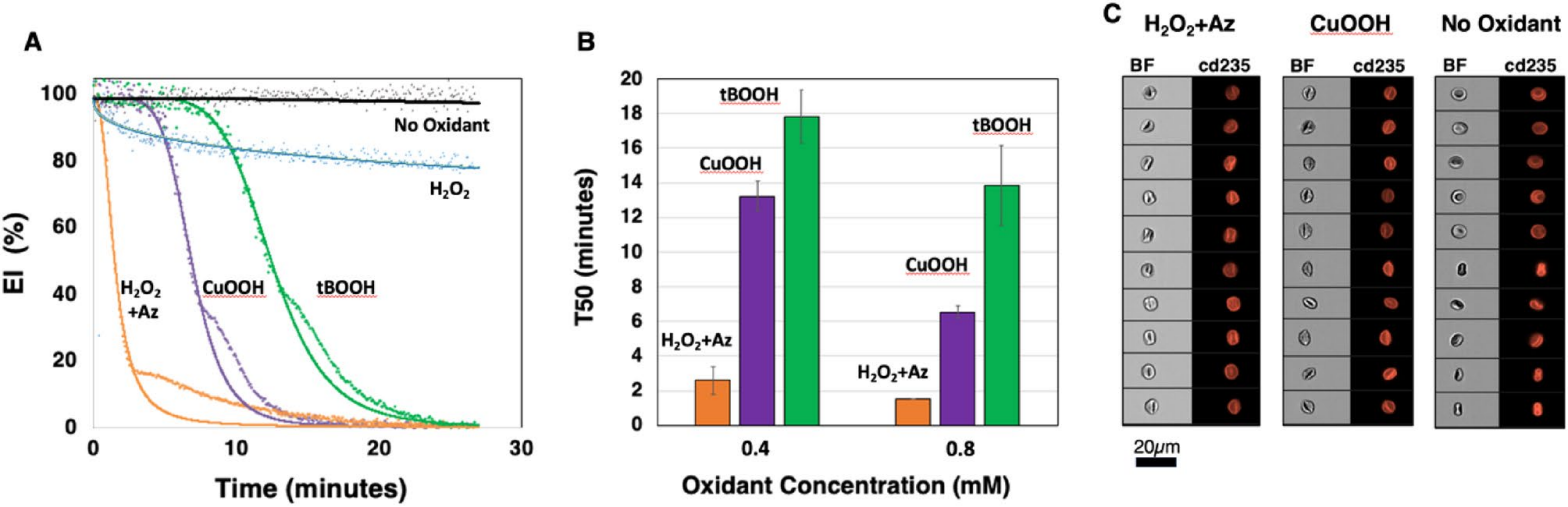
(**A**) Example curves of relative elongation index (EI%) of normal RBC exposed in the presence or absence of each oxidant at 0.8 mM with sigmoid cure overlayed onto the data points. (**B**) T50 for normal RBC samples exposed to each oxidant at 0.4 and 0.8 mM (n = 3). (**C**) Examples of the morphology of the RBC at the end of the incubation in the presence (H_2_O_2_ and azide_,_ or CuOOH) or absence (No Oxidant) of 0.8 mM oxidant with the brightfield (BF) image in the first column and anti-cd235 antibody labelling of the membrane in the second column.

Together these data show that oxidant stress under shear leads to a heterogeneous population of cells with different morphologies and orientation in the field of shear stress. We found that the time to reach the final equilibrium of EI depends on the concentration of the oxidant and the final (negative) level of EI on the type of oxidant. Figure 2A shows the change in EI for RBC incubated with or without 0.8 mM H_2_O_2_ (with or without azide), CuOOH, or tBOOH. The EI is not affected by azide alone (not shown). Pretreatment with azide inhibits the action of catalase and the EI drops rapidly when RBC were exposed to shear in the presence of H_2_O_2_. Data in Fig. 2B shows the comparison of the T50 of samples exposed to the three oxidants and that the decrease in deformability is dependent on the oxidant concentration in the range of 0.4–0.8 mM.

Table 1 shows a significant difference of the T50 between the oxidants at both concentrations. Whereas the concentration dependence of CuOOH is significant, the difference between 0.4 and 0.8 mM does not reach clear significance for tBOOH and H_2_O_2_ + Az.

**Table 1:** T50 values for normal RBC exposed to 0.4 or 0.8 mM of the three oxidants.

Oxidant	0.4 mM	0.8 mM	*p*-value
H_2_O_2_ + Az	2.6 ± 0.8	1.5 ± 0.0	0.074
CuOOH	13.2 ± 0.9	6.5 ± 0.4	< 0.001
tBOOH	17.8 ± 1.5	13.8 ± 2.3	0.066
p H_2_O_2_ + Az versus CuOOH	< 0.001	< 0.001	
p CuOOH versus tBOOH	0.010	0.006	
p tBOOH versus H_2_O_2_ + Az	< 0.001	0.001	

Figure 2C shows the morphology of typical examples of cells after exposure to shear stress with H_2_O_2_ + azide, CuOOH, or without oxidant (No Oxidant). The images show that in the absence of oxidant, virtually all cells retain their normal biconcave shape. The morphological changes caused by exposure to tBOOH (Fig. 1) and CuOOH (Fig. 2C) appeared slightly more extensive as compared to exposure to H_2_O_2_ and azide (Fig. 2C). Together these data show that the drop in EI is related to the ratio of oxidant to cell number, as well as the concentration and type of oxidant that the RBC is exposed to, probably reflecting the differences in targeted pathways and type of damage induced.

In addition to changes in morphology and behavior in the field of shear stress, hemolysis, fragmentation, and other membrane changes may occur. Samples were taken at the start, and end of the incubation in the viscometer when the EI had reached a minimum value. Hemoglobin was measured in the supernatant and expressed relative to the total hemoglobin present in the mixture to define hemolysis (Table 2). Cells challenged with these oxidants under shear in PVP did not result in hemolysis as cell free hemoglobin was less than 1% of all hemoglobin present. RBC exposed to shear stress in the absence of oxidant did not significantly shift mean cell volume (MCV) as measured after isovolumetric sphering^8^ (Table 2). Mean cellular hemoglobin (MCH) seemed to decrease using the Advia 2120, which did not correlate with the fact that little cell-free hemoglobin was present. The exposure to oxidant causes changes in the spectral characteristics of hemoglobin (supplementary Fig. 3) leading to the erroneous Advia 2120 derived MCH as the measurement of hemoglobin by the Advia 2120 is based on the normal spectroscopic characteristics.

**Table 2:** Hemolysis, mean cell volume (MCV) and mean cellular hemoglobin (MCH) content of untreated RBC (not exposed to oxidant or shear) and RBC treated with one of the oxidants at 0.6 mM in the RoxyScan assay.

Oxidant	Hemolysis (%)	MCV (fl)	MCH (pg)
Untreated	< 1	94.3 ± 0.9	30.3 ± 0.3
tBOOH	< 1	* 93.1 ± 1.0	12.7 ± 8.4
CuOOH	< 1	* 95.7 ± 1.1	8.8 ± 2.3
H_2_O_2_ + azide	< 2	* 93.4 ± 3.5	4.5 ± 4.9

Mean ± SD (n = 3). * *P* > 0.05 compared to untreated.

**Figure 3:**
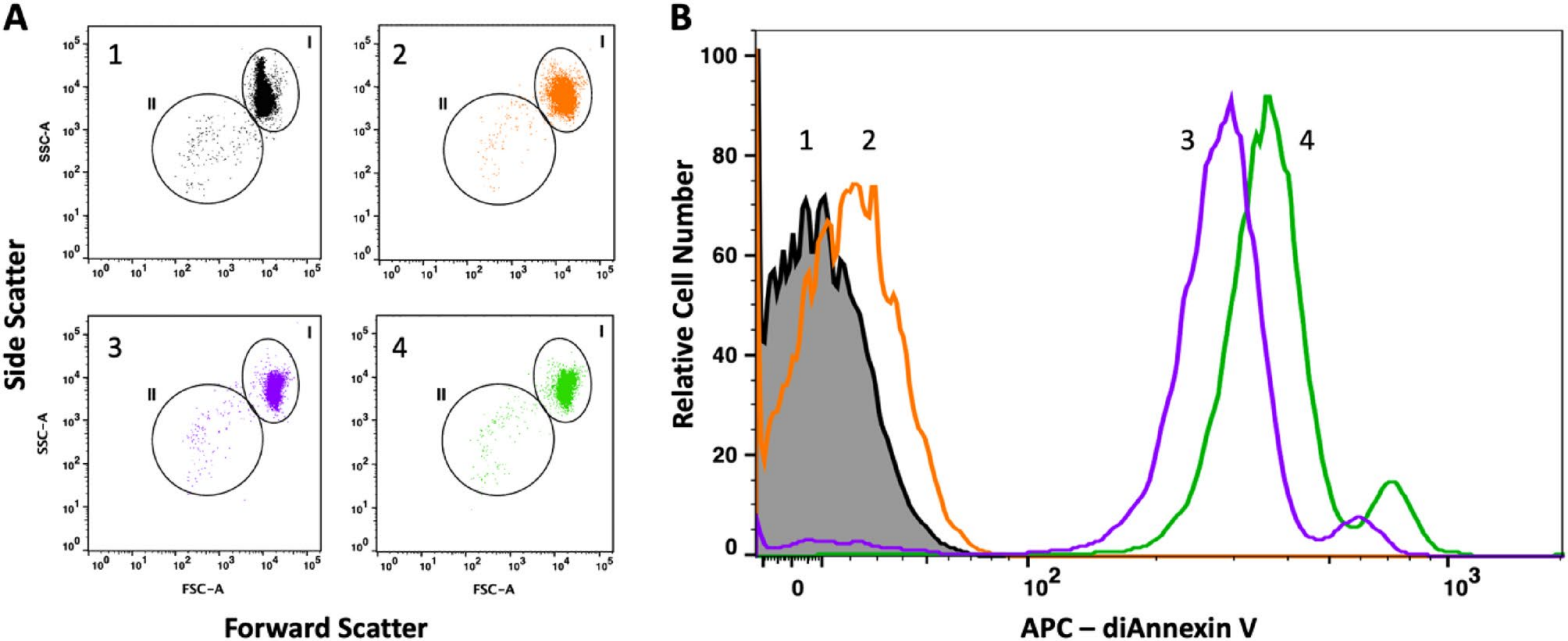
A typical flow cytometry assessment of RBC exposed to oxidant and shear. (**A**) Dot plots showing forward scatter on the x-axis and side scatter on the y-axis in the absence of oxidant (1) and after exposure to 0.8 mM H_2_O_2_ pretreated with azide (2), CuOOH (3), or tBOOH (4). The indicated regions of intact cells (I) and fragments (II) are shown for each plot. (**B**) Fluorescence histograms of APC-di-annexinV labeled RBC assayed without oxidant (1) or exposed to 0.8 mM H_2_O_2_ with azide (2), CuOOH (3), or tBOOH (4).

The flowcytometric assessment of RBC at the end of the RoxyScan assay is shown in Fig. 3. The dot plots in Fig. 3A show that exposure to oxidants under shear led to a shift in forward (FSC) and side (SSC) scatter of the intact RBC (gate I) compared to the control cells (plot 1). Oxidative stress caused by CuOOH (plot 3) or tBOOH (plot 4) resulted in the formation of very few fragments, and slightly increased by exposure to H_2_O_2_ with azide (plot 2). Labelling with fluorescent di-annexin V, showed less than 1% fluorescent events after exposure to shear in the absence of oxidant (Fig. 3B, peak 1). After exposure to shear and CuOOH (peak 3) or tBOOH (peak 4), most cells were fluorescently labeled, indicating a loss of phospholipid asymmetry and exposure of phosphatidyl serine (PS). In contrast, exposure of azide-treated cells to H_2_O_2_ under shear stress showed only a small percentage of fluorescent events (peak 2). However, all the fragments (from FSC/SSC gate II) were fluorescently labeled (not shown), indicating that in contrast to RBC, the fragments released from the H_2_O_2_ treated cells exposed PS.

RBC from SCD patients are less able to deal with oxidant stress^9–12^. To illustrate that RoxyScan would provide different data based on the source of blood, we explored the effect of oxidative stress on RBC of SCD patients. RoxyScan analysis was performed on 10 pairs of samples collected from 4 normal control donors and 10 different SCD subjects on different days as shown in Table 3. Each pair of normal and SCD samples was exposed to 0.6 mM TBOOH on the same day. The T50 was determined for each pair and a student’s T-test comparing the SCD and normal groups shows a significant difference (*p* < 0.05).

**Table 3:** RoxyScan T50 results of ten pairs of SCD and normal control samples exposed to 0.6 mM tBOOH.

Pair	T50 (min)	T50 (min)	%
	SCD	Normal	SCD
1	10.4	12.7	81.9
2	10.3	12.8	80.5
3	12.3	14.4	85.4
4	14.5	15.0	96.7
5	11.9	15.5	76.8
6	13.5	15.5	87.1
7	14.7	15.5	94.8
8	15.0	15.5	82.0
9	13.3	19.5	68.2
10	16.6	19.5	85.1
Avg	13.3	15.9	83.9
SD	2.0	2.5	8.3

The relative T50 of SCD blood as percentage of the T50 of normal blood is shown in the last column.

**Figure 4:**
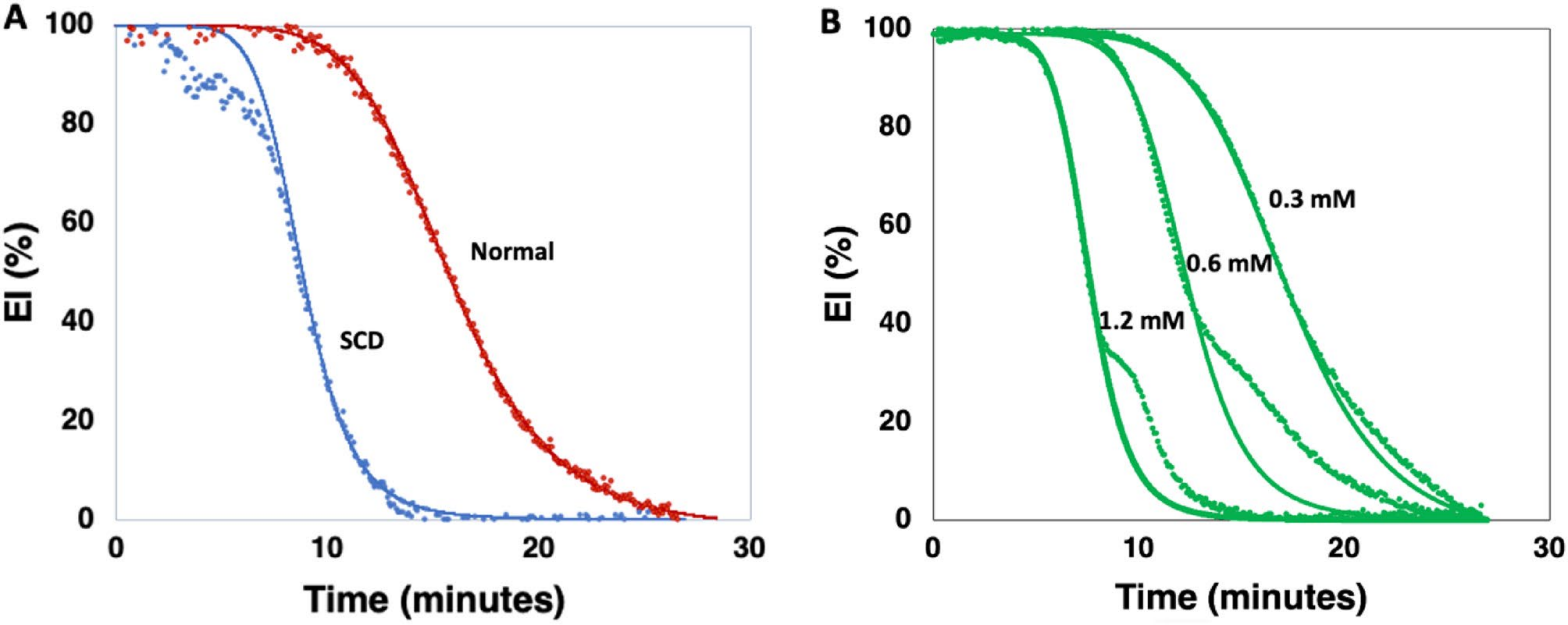
(**A**) Examples of RoxyScan curves of a SCD and a normal blood sample exposed to 0.6 mM tBOOH showing the sigmoid curve fit overlayed onto the data points. (**B**) RoxyScan curves from a normal blood sample incubated with 0.3 mM, 0.6 mM, and 1.2 mM tBOOH.

A number of SCD samples showed a pronounced drop in EI during the first minutes of incubation (Fig. 4A). This early decrease in EI varied between patients and suggests a more heterogeneous population in SCD blood with a subset that is less able to maintain its integrity under shear and oxidative stress. Data from these curves were not included in Table 3. We also observed a deviation from the sigmoid curve fit in some normal controls (Fig. 4B), which became more apparent at higher oxidative stress. The deviation of the sigmoid curve fit, found after the T50 is reached, suggested the presence of a subpopulation of cells, better equipped to withstand oxidative stress, or a different distribution in the heterogeneous population as shown in Fig. 1. The microscopic assessment of these samples in the viscometer failed to show a clear correlation between the number of altered cells in the population and the deviation of the sigmoid pattern.

## Discussion

Research from several labs has established the use of ektacytometry to define RBC characteristics over the last 5 decades^5–7^. Changes in RBC mechanical properties under shear and oxidative stress was reported before, using a custom-built instrument^13^. We used a commercially available instrument and defined this approach in detail under a limited set of conditions. The RoxyScan technology can be applied to evaluate the ability of different types of RBC to maintain their characteristics under shear and oxidative stress. Our data confirm similar studies that the shape change of the diffraction pattern does not exclusively reflect elongation of the RBC under these conditions^14,15^. The decrease of EI and the observed final negative EI value are due to a combination of changes in RBC morphology and the appearance of vertically oriented RBC in the field of shear stress. Normalization corrects for the different beginning and endpoints. Either of the values of T95, T50, or AUC can be used to describe the change in EI over time. The larger these numbers, the better the cell is able to maintain its viability under these conditions. These parameters hold potential to be used as biomarkers in various RBC disorders, which remains to be investigated.

RoxyScan experiments can be performed on whole blood or washed RBC, and the damage incurred in time depends on oxidant type, oxidant concentration, and the number of cells exposed to the oxidant. Millimolar concentrations of tBOOH induce changes in RBC deformability^16,17^, and our study adds to these observations by exploring the timeline of damage while RBC also experience shear stress. The ability of the RBC to deform in the field of shear stress is determined by intracellular viscosity: physicochemical properties of hemoglobin^18,19^ and membrane viscosity: elasticity of the spectrin cytoskeleton, and its interactions with transmembrane proteins and/or hemoglobin. When oxidative stress induces changes in hemoglobin and cytoskeletal proteins like spectrin^16,18,20–22^ the behavior of RBC in ektacytometry will be affected. There is compelling evidence that treatment of RBC with thiol-reactive molecules such as diamide^23^ or oxidants such as peroxides will strongly impair RBC deformability^16,24^ and change RBC shape^25,26^. The ratio of oxidant to RBC, rather than bulk oxidant concentration determines the loss of deformation, morphology, and behavior of individual RBC in the population. The damage to cells results in a reorientation in the field of shear as shown by the negative EI value.

Our data show little change in hemoglobin concentration, but we cannot exclude the effect of hemoglobin oxidation on intracellular viscosity given the apparent spectroscopic changes of hemoglobin under our conditions. The presence of methemoglobin showed that iron in the heme group changed from ferrous (Fe^2+^) to the ferric state (Fe^3+^), suggesting that the NADH-dependent enzyme methemoglobin reductase, responsible for converting methemoglobin back to hemoglobin, is not capable of fully performing this function, either overwhelmed by the level of hemoglobin oxidation, direct damage of the enzyme, or the lack of NADH. The exposure to H_2_O_2_ was used as an example to show the ability of RBC to maintain viability with a source of internally generated oxidative stress. SOD transfers superoxide to H_2_O_2_ which in turn is converted by catalase to water. The effective action of catalase is shown by the fact that 0.8 mM H_2_O_2_ only slightly decreased the EI in 25–30 min under shear stress. In contrast, pretreatment with azide, a catalase inhibitor^21^, followed by H_2_O_2_ led to a drastic drop in EI indicating the preeminent role of catalase to deal with H_2_O_2_ as was reported^21^, and underscores the eminent role of catalase to deal with the product of SOD^27^. Certainly, tBOOH and CuOOH do not report directly on stress experienced in the circulation but is likely similar to the stress by other ROS that are encountered by the RBC during its life.

In the absence of oxidant, the elongated shape of RBC under shear returned to a normal biconcave shape after the removal of the shear stress. In contrast, RBC exposed to oxidant and shear maintained their abnormal shape, indicating a loss of membrane flexibility due to oxidant damage of the membrane skeleton. The labeling with di-annexinV, indicating the loss of phospholipid asymmetry, indicates additional changes in the lipid bilayer of the RBC membrane when exposed to oxidative stress under shear.

The inability to deform, the change in morphology, and the loss of phospholipid asymmetry will likely lead to removal of such damaged RBC from the circulation in vivo*,* as is observed in many hereditary RBC disorders. The impaired ability of sickle RBC to deal with oxidative stress^28^, plays an important role in its drastically reduced lifespan. Our data, using the RoxyScan, confirms that sickle RBC are less able to maintain a proper redox status under oxidant and shear stress. No clinical data was available for these patients and did not provide a correlation between T50 and clinical conditions, such as genotype, treatment, or vaso-occlusive crises.

The EI reports on the RBC population as a whole and does not identify specific factors in individual RBC. The fact that the drop in EI can be fitted to a sigmoid pattern suggests a typical average and spread of a biological population of individual cells dealing with the stress on them. The presence of RBC that deviate from the population became apparent in blood collected from SCD patients. Whereas the T50 was decreased as compared to normal, indicating an average increased sensitivity to oxidative stress, some samples showed a deviation from the sigmoid curve fit at the start of the incubation, suggesting RBC in the population that were damaged more rapidly than the average cell. Our data do not allow assessment of what defines these deviant subpopulations. Nevertheless, each RBC population is a spectrum, ranging from cells that are just entering the circulation to cells that are at the point of removal. It is therefore reasonable to assume a distribution of sensitivities to stress.

In summary we suggest that the RoxyScan provides a simple way to monitor the overall ability of RBC to maintain mechanical characteristics with oxidative and shear stress in time, and may provide a potent assay to compare different oxidants and samples from different patients before and after treatment aimed to improve RBC viability.

## Methods

### Blood samples

All research was performed in accordance with relevant guidelines and regulations. The normal control blood samples were collected from 7 volunteer donors at the lab after informed consent according to the University of California, San Francisco (UCSF) IRB-approved protocol (#21-33332) for normal controls or received from unknown normal control donors (n = 21) provided after informed consent by Vitalant Research Institute (Denver, Co.). The SCD patient samples (n = 24) used for this study were anonymous discard samples obtained with UCSF IRB approval from the SCD clinic at UCSF Benioff Children’s Hospital in Oakland (BCHO) that included a waiver of Informed consent for use of anonymized samples. Because only anonymized patient samples were used, the SCD samples were not controlled for age, sex, disease state, treatment status, or any other lifestyle habits and no PHI was collected. There were no exclusion or inclusion criteria other than hemoglobin type. All SCD patient samples were from homozygous SCD (Hb SS) subjects and the normal control samples were collected from subjects known to be homozygous for normal hemoglobin (Hb AA). All blood samples, for SCD subjects and normal controls, collected into EDTA anticoagulant, were anonymized and de-identified.

### Reagents

Isotonic polyvinylpyrrolidone (PVP) at 294 ± 10 mOsm/kg, pH 7.4 ± 0.05, 30 ± 3 cP) was purchased from RR Mechatronics Manufacturing (BV Zwaag NL), or prepared locally (47 g/l PVP360, 8.4 mM sodium phosphate, 137 mM sodium chloride). The osmolarity was confirmed with a vapor pressure osmometer (Wescor, Logan, UT). Di-AnnexinV^29^ was labeled with Alexa-fluor 633 from Molecular Probes (Eugene, OR) according to manufacturers’ instructions. The Dako phycoerythrin (PE)—labeled monoclonal mouse-anti human glycophorin-A antibody (anti-cd235a, clone JC159) was purchased from Agilent (Santa Clara, CA). All other reagents were purchased from Sigma-Aldrich (St. Louis, MO) unless otherwise noted. Hepes buffered saline (HBS, 290 ± 5 mOsmol, pH7.4) was prepared from 10 mM Hepes and 145 mM sodium chloride. A fixative solution (2% paraformaldehyde/0.5% glutaraldehyde) was prepared in HBS. The manufacturer’s specified concentrations were used to create stock solutions of three different oxidants by dilution to 100 mM in HBS: tert-butyl hydroperoxide (tBOOH), cumene hydroperoxide (CuOOH), and hydrogen peroxide (H_2_O_2_) with sodium azide. Oxidant dilutions in PVP were prepared from the concentrated stocks. Sodium azide, when used in experiments was added to a final concentration of 1 mM from the concentrated stock.

### Instrumentation

**Figure 5:**
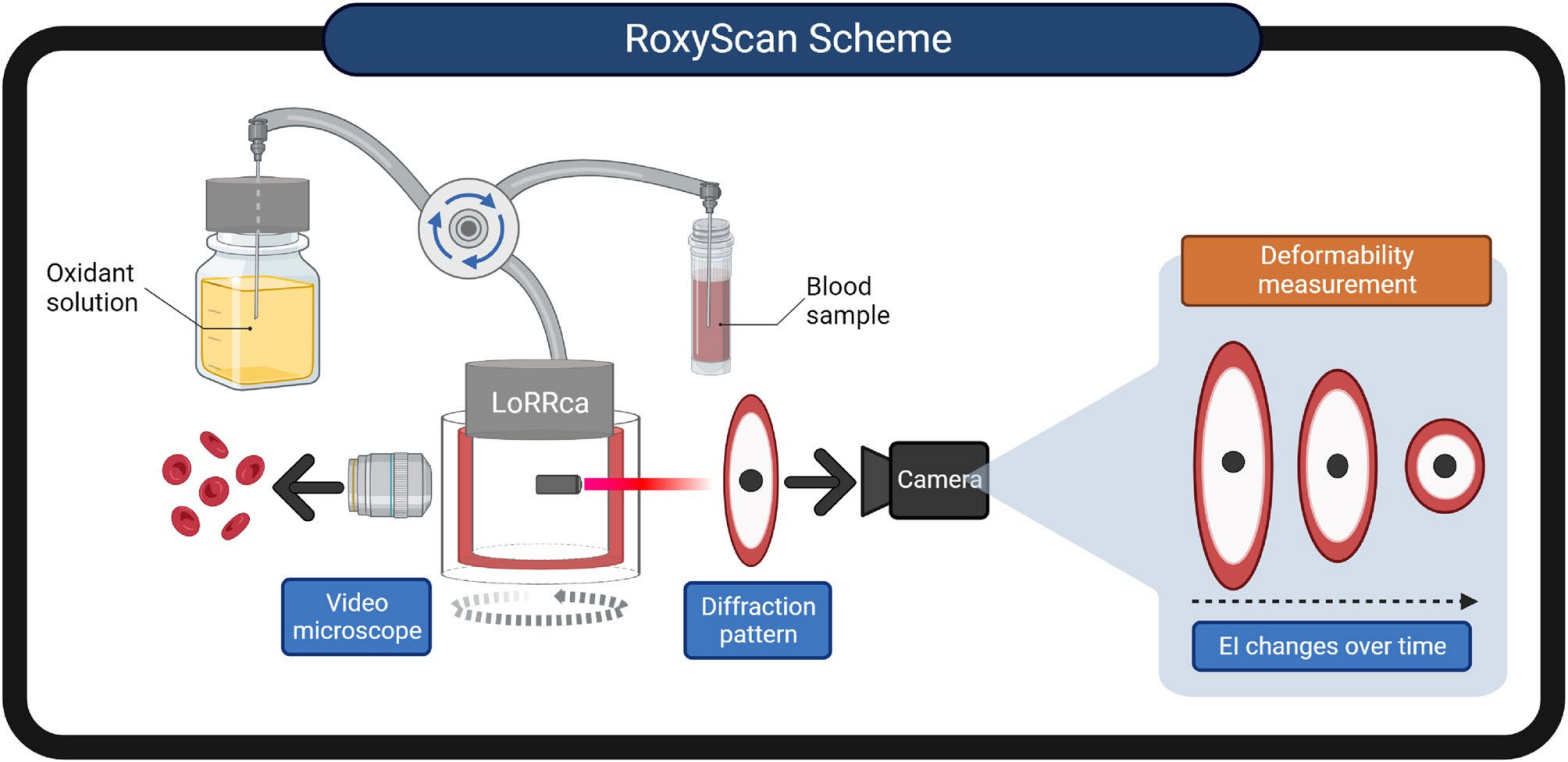
Schematic of the RoxyScan assay setup using the LoRRca MaxSis ektacytometer showing the mixing of the oxidant in isotonic PVP with the blood sample in isotonic PVP. The LoRRca components (laser (red arrow), camera, and video microscope) and the resulting blood cell images and diffraction patterns allow the deformability measurement by elongation index (EI). Figure 5 was created with BioRender.com.

The Advia 2120 (Siemens Healthineers, Mountain View, CA) was used to characterize RBC and determine the necessary blood volumes to standardize the number of cells in the experiments. The Fortessa flowcytometer (Becton Dickinson, San Jose, CA) was used to monitor fluorescent labelling of the intact and fragmented RBC. The Amnis ImageStream Mk II imaging flow cytometer (Cytek, Boston. MA) was used to visualize the morphology and fluorescence of RBC. The LoRRca MaxSis ektacytometer (RR Mechatronics Manufacturing BV Zwaag NL)^30^, was used to obtain the osmotic deformability profile (supplementary Fig. 4) and also allowed addition of cells into the viscometer as shown in Fig. 5 for the RoxyScan assay as described below. The Cary-50 Bio UV–visible spectrophotometer (Varian, Houston, TX) was used to determine the change in the hemoglobin spectrum of cells after the RoxyScan assay (Supplementary Fig. 3).

### RoxyScan assay

Isotonic PVP with or without oxidant (0.3–1.2 mM) was mixed (3 + 1, V + V) with 200 × 10^6^ RBC/ml in isotonic PVP by a pump and three-way valve into the LoRRca viscometer. The concentration range of oxidant was determined by those that caused a significant change within a reasonable amount of time (25–30 min). A shear stress of 30 ± 3 mPa-s, the typical range for osmotic deformability measurements^6,7,31^, set by the speed of the viscometer and the viscosity of the PVP, was used to create maximal deformation under isotonic conditions^30^. The pump transports RBC within 10 s to the path of the laser. The LoRRca software translates the diffraction pattern into the elongation index (EI)^7^. The video microscopic interface assesses shape and behavior of RBC in the viscometer. All experiments were performed at 37 °C.

### RBC assessment

RBC samples (~ 50 × 10^6^ cells/ml) from the viscometer were collected and immediately diluted 2.5-fold with HBS followed by centrifugation at 1000xg to pellet the cells and remove the PVP and oxidant. Total hemoglobin in the mixture and cell free hemoglobin in the supernatant was determined using Drabkin's reagent according to the manufacturer’s protocol (Sigma-Aldrich, St. Louis, MO). The pellet was resuspended in 1 ml HBS or for assessment with spectroscopy, flow cytometry, and Advia 2120 analysis.

### PS exposure

For flowcytometric analysis of RBC phospholipid rearrangement, the RBC pellet (50 × 10^6^ cells) was resuspended in 1 ml of HBS containing 2 mM CaCl_2_. Aliquots (0.1 ml) of RBC (5 × 10^6^ cells were incubated with 10 ul Alexa Fluor (AF) 633 diAnnexinV (1 µg/ml) in 1 ml of HBS containing 2 mM CaCl_2_ for 30 min at room temperature. After incubation, RBC were pelleted by centrifugation for 3–5 min at room temperature at 1000 × g. The supernatant was removed by aspiration and the cells were resuspended in HBS with 2 mM CaCl_2_ at ~ 10^6^ cells/ml prior to Fortessa analysis. Intact RBC were gated on the forward scatter versus side scatter dot plot. Alexa Fluor (AF) 633 is a far-red dye with excitation at 633 nm and emission is detected at 670 ± 15 nm. The relative fluorescence of the AF633 diAnnexin V labeled RBC is presented as histograms. Annexin-positive populations were gated against a sample of unstained cells^29^.

### Image analysis

For imaging of morphology changes, the RBC pellet (25–50 × 10^6^ cells) was resuspended in 1 ml of fixative solution and incubated for 10–12 min at room temperature while mixing. After fixation, cells were pelleted by centrifugation for 3–5 min at room temperature at 1000 × g. The fixative was removed by aspiration and the cells were resuspended in HBS at 10^6^ cells/ml and incubated for 30 min at room temperature with 5 µl anti-cd235a PE. After antibody incubation, RBC were pelleted by centrifugation for 3–5 min at room temperature at 1000 × g. The HBS was removed by aspiration and the cells were resuspended in HBS at 2 × 10^7^ cells/ml prior to ImageStream analysis within 2 h of cell preparation. A minimum of 10,000 events were acquired for each sample. PE fluorescence was excited with the 488 laser and the emission (578 nm) was detected in channel 3 (560–596 nm). RBC are gated on the area versus aspect ratio dot plot. Brightfield and fluorescent images were used to visualize any shape change caused in the RoxyScan assay.

### Supplementary Information

Supplementary Figures.

## Data Availability

The datasets generated during and/or analysed during the current study are available from the corresponding author upon reasonable request.
