# Supplementary material for: The RoxyScan is a novel measurement of red blood cell deformability under oxidative and shear stress
Source: Sci Rep. 2024 Mar 15;14:6344. doi: 10.1038/s41598-024-56814-8 (PMC10943210; doi:10.1038/s41598-024-56814-8)
Supplement: Supplementary file 1 — Supplementary Figures. [file 41598_2024_56814_MOESM1_ESM.docx]

**
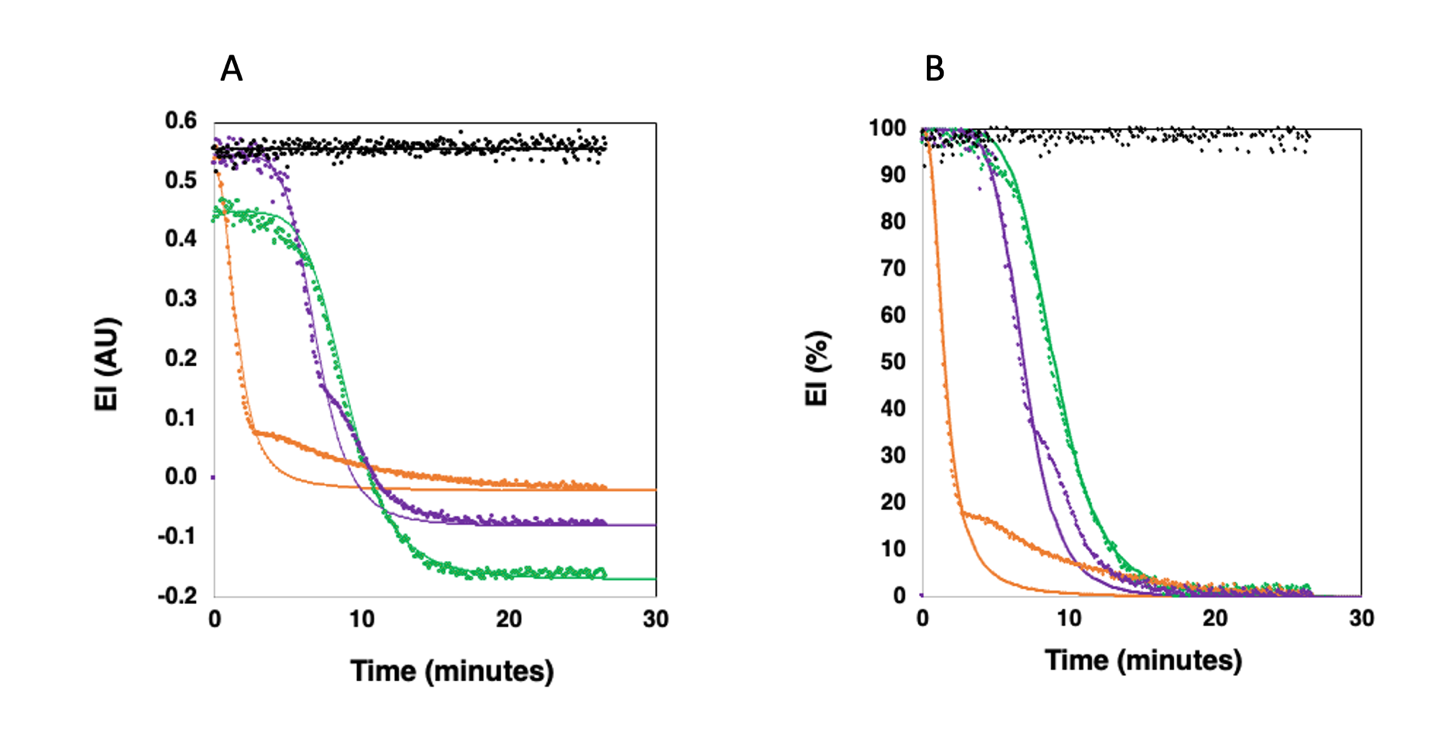
**

**Supplementary Figure 1**: RoxyScan results of 4 different samples processed in the presence or absence of oxidants. A: raw data overlayed with sigmoid curve fit of H_2_O_2_ in orange, CuOOH in purple, tBOOH in green, and no oxidant in black. B: the same data shown in A but normalized to the start EI and end EI for each oxidant and control (no oxidant).

**
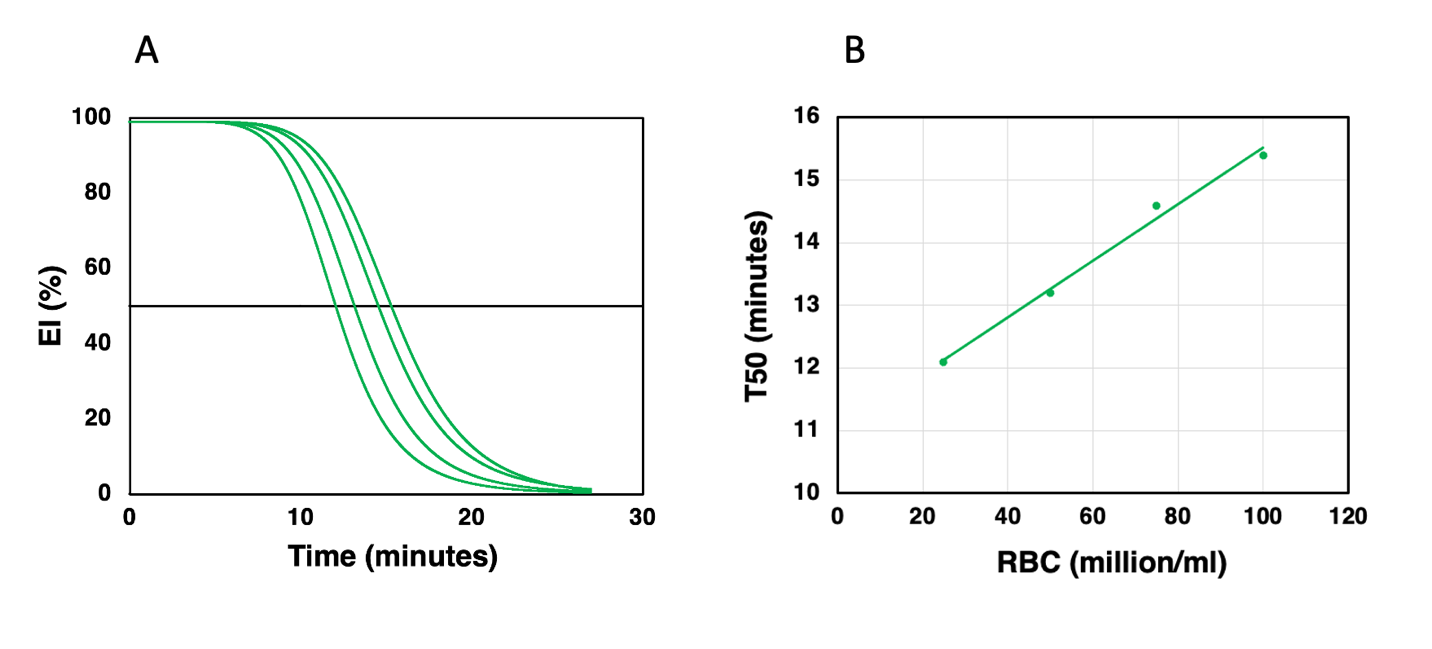
**

**Supplementary Figure 2: A:** RoxyScan curves of different cell concentrations (25, 50, 75 or 100 million RBC/ml) of normal RBC exposed to 0.6 mM tBOOH and the same shear stress. **B:** The t50 values from the curves in A show a direct correlation to the RBC count.


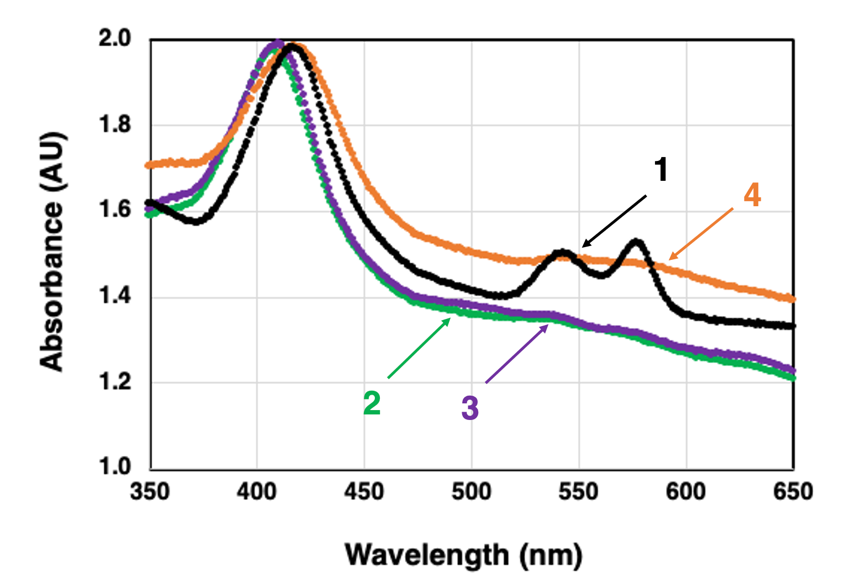


**Supplementary Figure 3:** Spectrum of hemoglobin after exposure of RBC to shear stress without oxidant (1) and with 0.6 mM tBOOH (2), CuOOH (3), H_2_O_2_ with azide (4). Before exposure to shear and oxidant stress, the hemoglobin spectrum shows the typical maxima of oxygenated hemoglobin at 415nm and the doublet between 500 and 600 nm (1). After incubation, under shear stress, in the presence of tBOOH, CuOOH, or H_2_O_2_+Az, the spectrum changes dramatically, indicating oxidation of hemoglobin and the presence of methemoglobin (2,3,4).


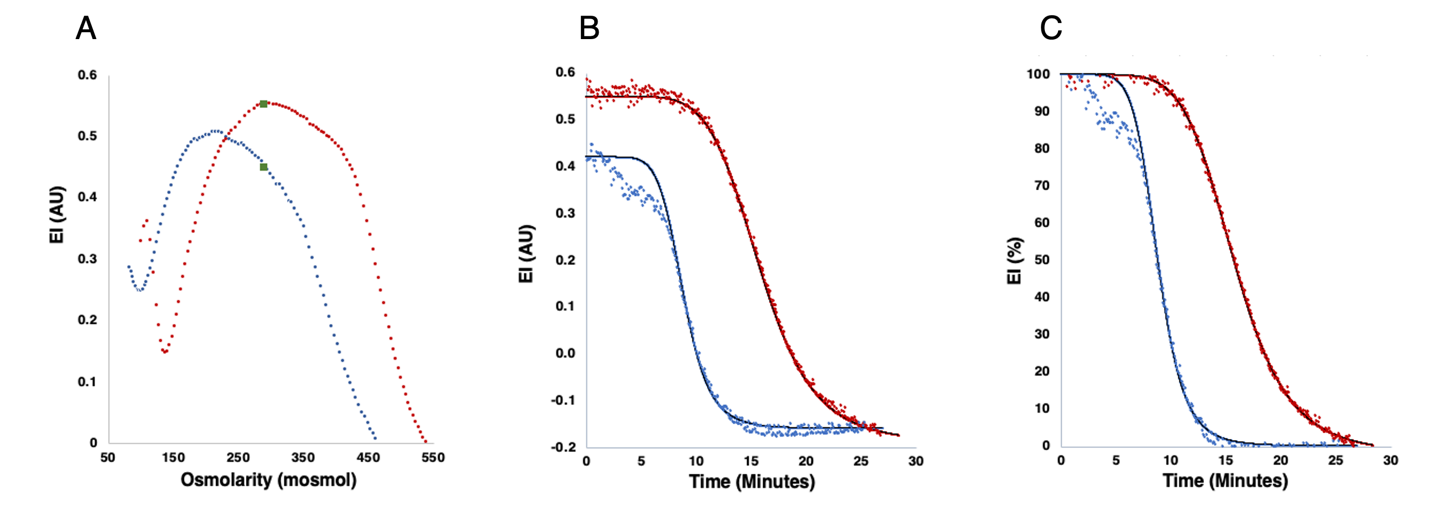


**Supplementary Figure 4**. **A**: Osmotic deformability profile of a SCD sample (blue) and normal control (red). The elongation index (EI) at 290 mosmol is maximal for normal control, but lower for the sickle cell sample. **B**: RoxyScan data of the cells shown in A exposed to 0.6mM tBOOH at 290 mosmol showing starting EI = 0.45AU for the SCD sample in blue or 0.55 AU for the normal control in red. **C**: Normalized curves from the data as shown in B from starting EI (100%) to final EI (0%) and the sigmoid curve fit used to obtain the T50 values, 8.8 minutes for the SCD sample and 15.8 minutes for the normal control.
